# A novel conditional survival nomogram for monitoring real-time prognosis of non-metastatic triple-negative breast cancer

**DOI:** 10.3389/fendo.2023.1119105

**Published:** 2023-02-24

**Authors:** Xiangdi Meng, Yuanyuan Cai, Xiaolong Chang, Yinghua Guo

**Affiliations:** Department of Radiation Oncology, Weifang People’s Hospital, Weifang, China

**Keywords:** triple-negative breast cancer, conditional survival, nomogram, prognostic factor, overall survival

## Abstract

**Background:**

Conditional survival (CS) is defined as the possibility of further survival after patients have survived for several years since diagnosis. This may be highly valuable for real-time prognostic monitoring, especially when considering individualized factors. Such prediction tools were lacking for non-metastatic triple-negative breast cancer (TNBC). Therefore, this study estimated CS and developed a novel CS-nomogram for real-time prediction of 10-year survival.

**Methods:**

We recruited 32,836 non-metastatic TNBC patients from the Surveillance, Epidemiology, and End Results (SEER) database (2010-2019), who were divided into training and validation groups according to a 7:3 ratio. The Kaplan-Meier method estimated overall survival (OS), and the CS was calculated using the formula CS(y|x) =OS(y+x)/OS(x), where OS(x) and OS(y+x) were the survival of x- and (x+y)-years, respectively. The least absolute shrinkage and selection operator (LASSO) regression identified predictors to develop the CS-nomogram.

**Results:**

CS analysis reported gradual improvement in real-time survival over time since diagnosis, with 10-year OS updated annually from an initial 69.9% to 72.8%, 78.1%, 83.0%, 87.0%, 90.3%, 93.0%, 95.0%, 97.0%, and 98.9% (after 1-9 years of survival, respectively). The LASSO regression identified age, marriage, race, T status, N status, chemotherapy, surgery, and radiotherapy as predictors of CS-nomogram development. This model had a satisfactory predictive performance with a stable 10-year time-dependent area under the curves (AUCs) between 0.75 and 0.86.

**Conclusions:**

Survival of non-metastatic TNBC survivors improved dynamically and non-linearly with survival time. The study developed a CS-nomogram that provided more accurate prognostic data than traditional nomograms, aiding clinical decision-making and reducing patient anxiety.

## 1 Introduction

Triple-negative breast cancer (TNBC) is the most aggressive molecular subtype of breast cancer, accounting for 10-20% of all cases with a poor prognosis (1–7). Statistically, the postoperative recurrence rate of this disease was as high as 25% (5), the 5-year overall survival (OS) for regional tumors was about 64%-72% (1, 2, 4, 7–9), and the median survival time after metastasis was only 13.3 months (5). However, as valuable as these data may be at initial diagnosis, they may be inaccurate for survivors. Traditional survival analysis can only use the patient’s status at the initial diagnosis as an assessment landmark and never consider how long the patient has survived (10, 11). Such static evaluations were unfortunate for patients who survived for several years, as no improvement in survival was observed during long-term follow-up. Previous studies demonstrated that the prognosis of long-term survivors improved dynamically over time (10–13). With improved treatment strategies, survivors achieved long-term survival and might prefer to know accurate real-time prognostic information during ongoing follow-up.

More recently, results from the M.D. Anderson Cancer Center suggested that the risk of recurrence and death in non-metastatic TNBC was strongly time-dependent, but no further studies (7). Conditional survival (CS) analysis conveyed the dynamics of survival rates over time which could provide a real-time updated estimate of survival. It was defined as the probability that a patient would survive for a further y years after surviving for x years after diagnosis (10, 11). Currently, CS analysis has been widely used in many cancers (e.g., colorectal cancer (12, 13), stomach cancer (14), liver cancer (15), esophageal cancer (16), nasopharyngeal cancer (17), lymphoma (18), pancreatic cancer (19), etc.) to assist physicians in optimizing clinical decisions and significantly reduce the psychological distress of survivors. However, to our knowledge, although many TNBC prognostic assessment tools have been developed (9, 20–27), they have never focused on prognostic changes over time. In addition, the common nomograms, while allowing for individualized prediction, have never provided dynamic prognostic information over survival time.

Considering the significant differences in clinicopathological characteristics, treatment strategies, and prognosis between metastatic TNBC and stage I-III TNBC, a subset of patients with non-metastatic TNBC was selected for our study. The aim of this study was to reveal the change in survival of non-metastatic TNBC patients over time and to develop the first CS-nomogram to provide clinicians and patients with personalized and real-time updated dynamic prognostic information.

## 2 Material and methods

### 2.1 Data sources, patient selection, and variables

This study enrolled patients in the Surveillance, Epidemiology, and End Results (SEER) database diagnosed with triple-negative breast cancer between 2010 and 2019 [the International Classification of Diseases for Oncology (ICD-O-3) site recode: “Breast” and morphology code: “8500/3” with negative receptor status for estrogen receptor (ER) status, progesterone receptor (PR) status, human epidermal growth factor receptor 2 (Her 2)] (https://seer.cancer.gov/). Before using this database, we signed the SEER Research Data Use Agreement and the Best Practices Assurance and accessed the data *via* login 11578-Nov2021. So, the institutional ethics committee exempted the study from ethical review.

The 53,253 patients recruited were selected on the basis of the following exclusion criteria (1): Distant metastasis or metastatic status unknown; (2) Unconfirmed by positive histopathology; (3) Not the first primary malignancy; (4) Age <18 years old; (5) required variables were unknown; (6) The follow-up time was 0 months. The AJCC 7th and 8th editions of breast cancer used in this study were consistent and could therefore be combined. The final selected patients were divided into training and validation groups according to the ratio of 7:3. Age at diagnosis, marital status, race, tumor stage, histological grade, and treatment may be related to the prognosis of TNBC and were used in this study.

#### 2.1.1 Statistical analysis

The number and proportion of categorical variables were recorded. In addition, means and standard deviations (SDs) were reported for continuous variables that followed a normal distribution; otherwise, medians and interquartile ranges (IQRs) were reported. The clinical endpoint of the study was OS, defined as the time from the patient’s disease diagnosis to death and estimated using the Kaplan-Meier method.

CS was calculated by CS(y|x) =OS(y+x)/OS(x). CS(y|x) was the probability that a patient would survive an additional y years after having survived x years from initial diagnosis. Furthermore, OS(x) and OS(y+x) were the Kaplan-Meier estimated patient survival rates for x- and (x+y)-years, respectively. For example, if the patient has survived 3 years after the diagnosis and wants to estimate the probability of surviving another 2 years, the result was calculated as CS(2|3) = OS(2 + 3)/OS(3) =5-year OS rate/3-year OS rate. Additionally, annual hazard rates assessed mortality in patients followed up over one year and were used to interpret and reinforce the conclusions conveyed by the survival curves.

For variable selection, we used the least absolute shrinkage and selection operator (LASSO) regression with 10-fold cross-validation to avoid over-fitting. Meanwhile, multivariate Cox regression was used to confirm the effect of the selected predictors on survival and to develop a nomogram. Finally, the CS concept was applied to the nomogram to construct a CS-nomogram for individualized prediction of real-time prognosis updated over time. It quantified patient risk and used risk scores to obtain individualized survival and CS rates. Calibration curves assessed the accuracy of the CS-nomogram. The closer the curve was to the 45° line, the more accurate the model was. Discrimination and stability were estimated using the concordance index (C-index) and time-dependent area under the curve (AUC). We calculated the AUC for 1-10 years to confirm that the model’s performance did not deteriorate over time. Finally, the decision curve analysis (DCA) validated the clinical utility by calculating the net benefit of triggering a medical intervention using our CS-nomogram. The statistics of this study were analyzed using R (version 4.1.0). In the two-tailed test, P values less than 0.05 were considered statistically significant.

## 3 Results

### 3.1 Clinicopathological characteristics

**Figure 1:**
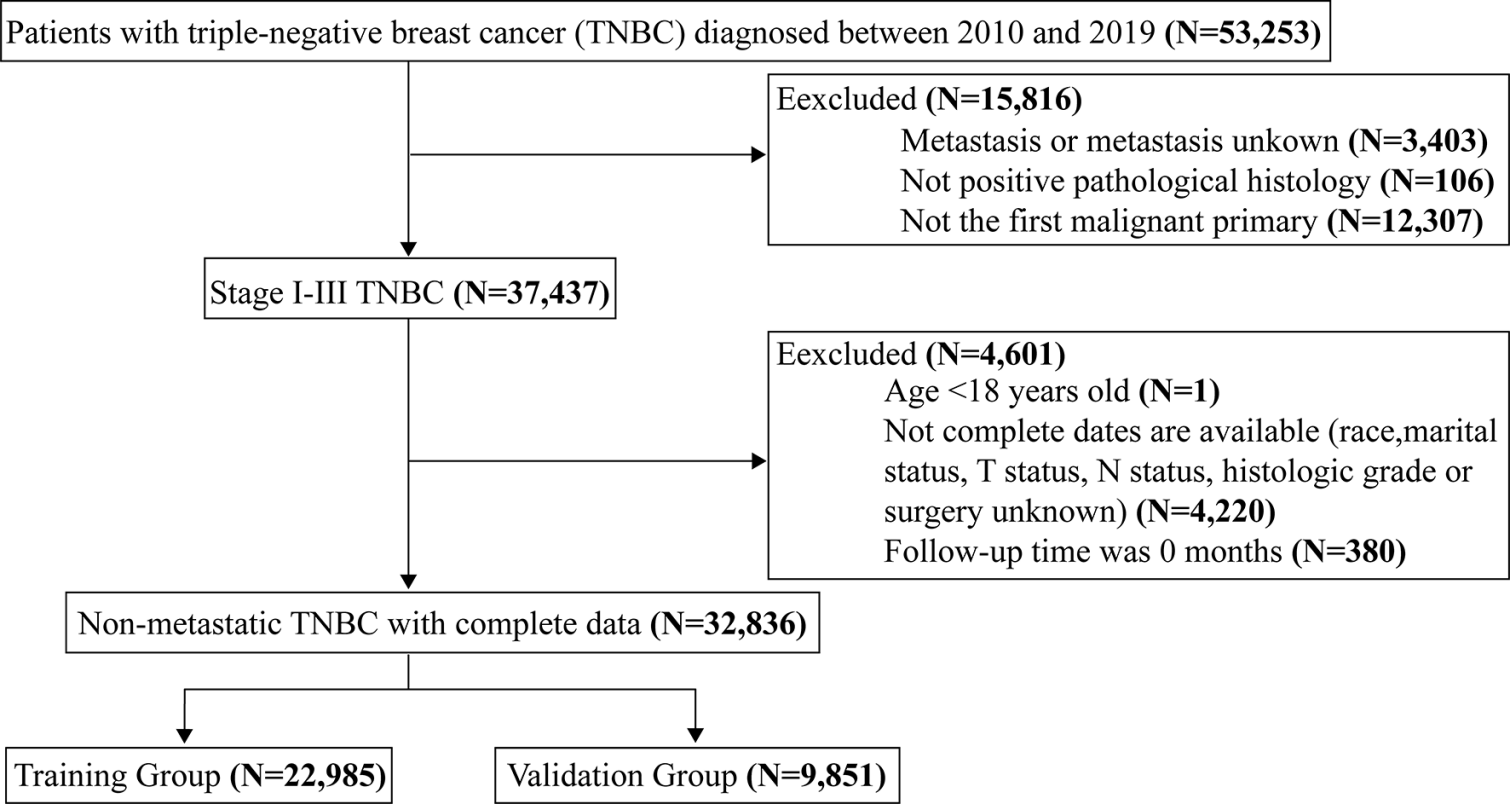
Flow chart for screening patients with non-metastatic triple-negative breast cancer.

A total of 32,836 women diagnosed with non-metastatic TNBC between 2010 and 2019 were screened for this study, of whom 22,985 were included in the training group and 9,851 in the validation group (**Figure 1**). The mean age of the whole cohort was 57.3 years (SD =14 years), and the majority were in stage I-II at diagnosis, with 15.3% (5,019/32,856) in stage III. Regarding treatment, the vast majority underwent surgery [51.3% for breast-conserving surgery (BCS) and 43.5% for mastectomy] and chemotherapy in 79.4% (26,078/32,836) of patients. In addition, 6,057 (18.4%) deaths were recorded over 10 years, with a median study follow-up of 41 months (IQR: 19 months, 74 months). See **Table 1** for details.

**Table 1:** Clinicopathologic characteristics of non-metastatic triple-negative breast cancer patients.

Characteristics	Whole cohort	Training group	Validation group
	n=32,836 (%)	n=22,985 (%)	n=9,851 (%)
Age at diagnosis, years			
Mean (SD)	57.3 (14.0)	57.3 (14.0)	57.5 (13.9)
18-39	3941 (12.0)	2790 (12.1)	1151 (11.7)
40-70	23005 (70.1)	16079 (70.0)	6926 (70.3)
>70	5890 (17.9)	4116 (17.9)	1774 (18.0)
Marital status			
Unmarried	14025 (42.7)	9861 (42.9)	4164 (42.3)
Married	18811 (57.3)	13124 (57.1)	5687 (57.7)
Race			
Black	6577 (20.0)	4625 (20.1)	1952 (19.8)
White	23432 (71.4)	16368 (71.2)	7064 (71.7)
Other	2827 (8.6)	1992 (8.7)	835 (8.5)
T status (AJCC 8^th^)			
T1	14217 (43.3)	9918 (43.1)	4299 (43.6)
T2	14547 (44.3)	10191 (44.3)	4356 (44.2)
T3	2641 (8.0)	1857 (8.1)	784 (8.0)
T4	1431 (4.4)	1019 (4.4)	412 (4.2)
N status (AJCC-8^th^)			
N0	21742 (66.2)	15289 (66.5)	6453 (65.5)
N1	7996 (24.4)	5551 (24.2)	2445 (24.8)
N2	1759 (5.4)	1193 (5.2)	566 (5.7)
N3	1339 (4.1)	952 (4.1)	387 (3.9)
TNM (AJCC-8^th^)			
I	11584 (35.3)	8111 (35.3)	3473 (35.3)
II	16233 (49.4)	11364 (49.4)	4869 (49.4)
III	5019 (15.3)	3510 (15.3)	1509 (15.3)
Histological grade			
I	5932 (18.1)	4199 (18.3)	1733 (17.6)
II	26689 (81.3)	18645 (81.1)	8044 (81.7)
III-IV	215 (0.7)	141 (0.6)	74 (0.8)
Surgery			
No	1703 (5.2)	1210 (5.3)	493 (5.0)
BCS	16841 (51.3)	11769 (51.2)	5072 (51.5)
Mastectomy	14292 (43.5)	10006 (43.5)	4286 (43.5)
Chemotherapy			
No	6758 (20.6)	4759 (20.7)	1999 (20.3)
Yes	26078 (79.4)	18226 (79.3)	7852 (79.7)
Radiotherapy			
No	15219 (46.3)	10640 (46.3)	4579 (46.5)
Yes	17617 (53.7)	12345 (53.7)	5272 (53.5)

AJCC-8th, American Joint Committee on Cancer (8th Edition); TNM, tumor-node-metastasis; BCS, breast conservation surgery; SD, standard deviation.

### 3.2 Conditional survival analysis

**Figure 2:**
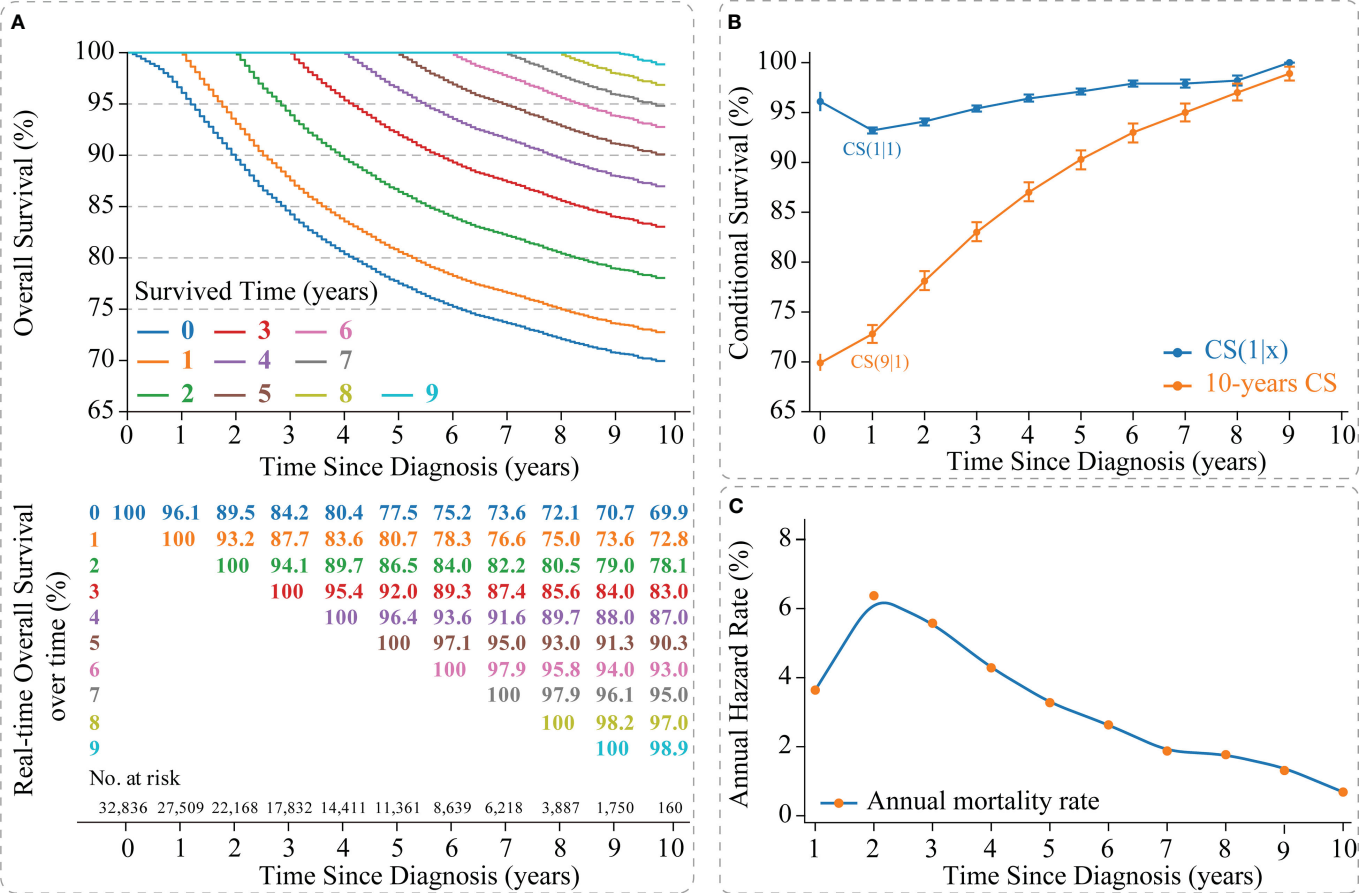
Survival analysis of non-metastatic triple-negative breast cancer. **(A)** Kaplan–Meier curves estimating real-time survival after surviving for 0-9 years; **(B)** CS(1|x) curve showing the probability of survival another year after surviving for x years since diagnosis and 10-year CS curve showing the 10th year of survival after surviving for x years since diagnosis; **(C)** Annual hazard rate curve. CS, conditional survival.

The Kaplan-Meier method estimated an OS of 77.5% [95% confidence interval (CI): 0.769-0.780] and 69.9% (95% CI: 0.691-0.708) at 5- and 10-years, respectively, for non-metastatic TNBC patients (**Figure 2A**). CS analysis found that patients’ real-time OS gradually improved over time. For each additional year a patient survived after diagnosis, her 10-year survival rate increased, which gradually updated from an initial 69.9% to 72.8%, 78.1%, 83.0%, 87.0%, 90.3%, 93.0%, 95.0%, 97.0%, and 98.9% (after surviving for 1-9 years, respectively) (**Figures 2A, B**).

Meanwhile, we found that survival improvement over time was nonlinear. The CS(1|x) curve showed the survival rate for the next year after the patient survived x years, in which the 2nd year survival rate for patients who survived 1 year after diagnosis showed the lowest survival rate [CS(1|1) =93.2% (95% CI: 92.9%-93.5%)] (**Figure 2B**). In addition, the annual hazard curve reported the highest mortality rate for non-metastatic TNBC patients in the second year after diagnosis (**Figure 2C**).

### 3.3 Development and validation of the CS-nomogram

**Figure 3:**
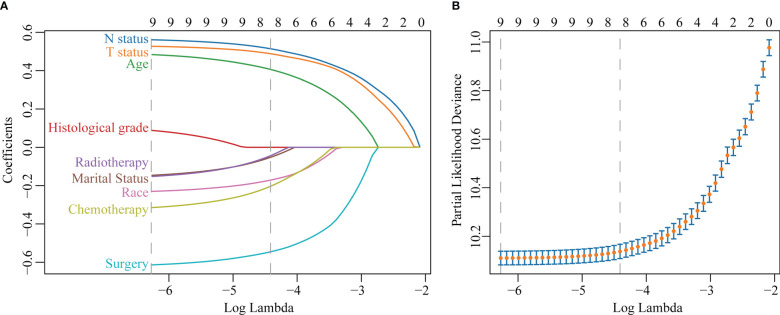
Predictor screening. **(A)** The least absolute shrinkage and selection operator (LASSO) regression and **(B)** 10-fold cross-validation.

**Figure 4:**
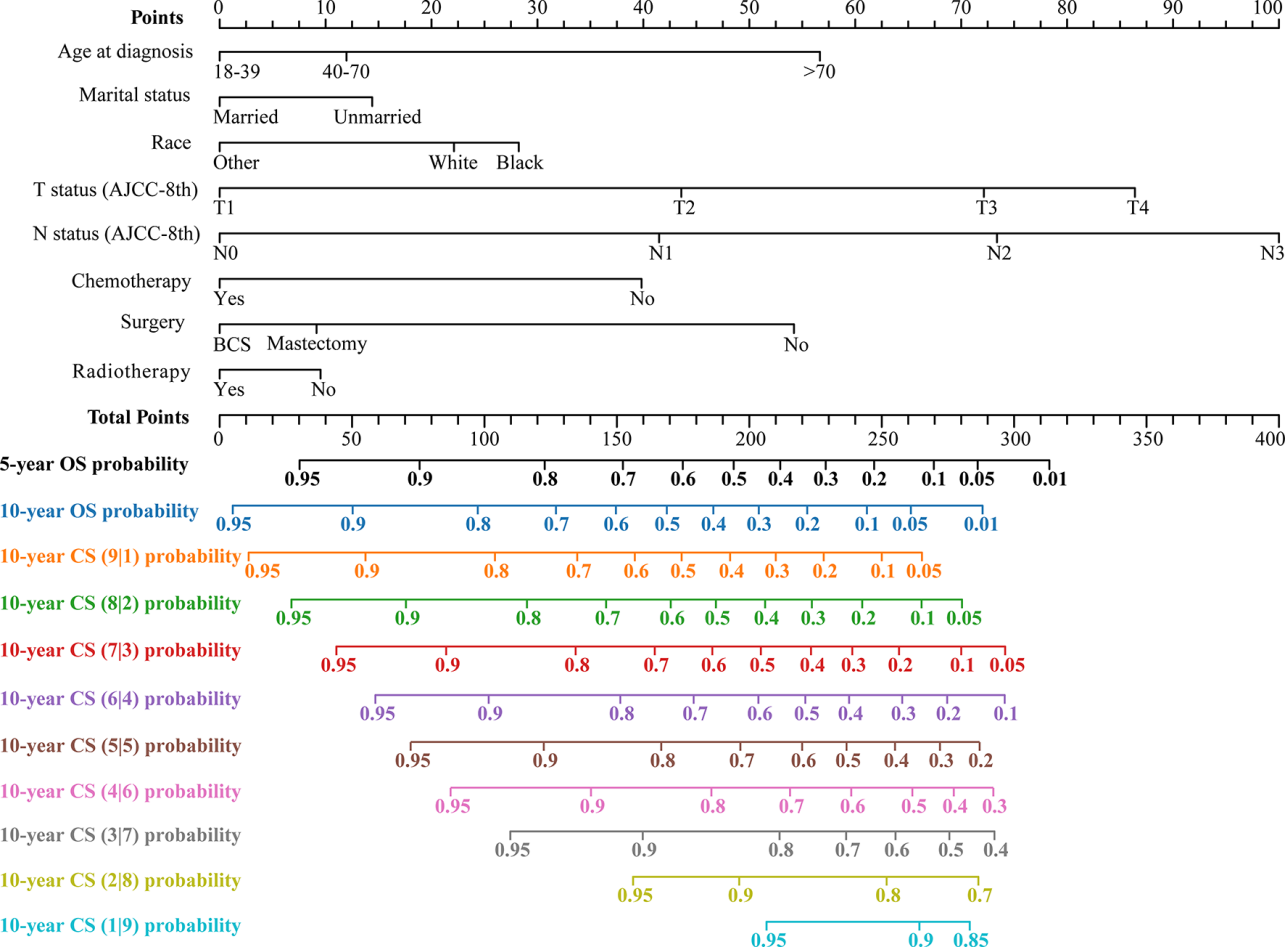
Conditional survival nomogram (CS-nomogram) predicting 5- and 10-year overall survival (OS) and 10-year conditional survival (CS) for non-metastatic triple-negative breast cancer. AJCC-8th, American Joint Committee on Cancer (8th Edition); BCS, breast conservation surgery.

The LASSO regression with 10-fold cross-validation selected eight predictors (age, marital status, race, T status, N status, chemotherapy, surgery, and radiotherapy) for developing the prognosis model (**Figures 3A, B**). Multivariate Cox regression forest plot showed that these predictors were significantly associated with survival in non-metastatic TNBC patients (P<0.001, **Supplementary Figure 1**), which were used to develop a nomogram. This model quantified risk to estimate prognosis individually. After calculating the patient’s 1-10 years survival rates, we applied the CS formula to the model to develop the CS-nomogram, which could update the patient’s 10-year OS in real-time with survived time (**Figure 4**). The CS-nomogram quantified the entered patient variables as points, and the sum of the points corresponded to her 5-year, 10-year OS and 10-year CS. For example, a patient with a total risk score of 200 had a 10-year OS of about 32% at diagnosis, but if she survived for 3 years, the 10-year OS was adjusted to 51%.

**Figure 5:**
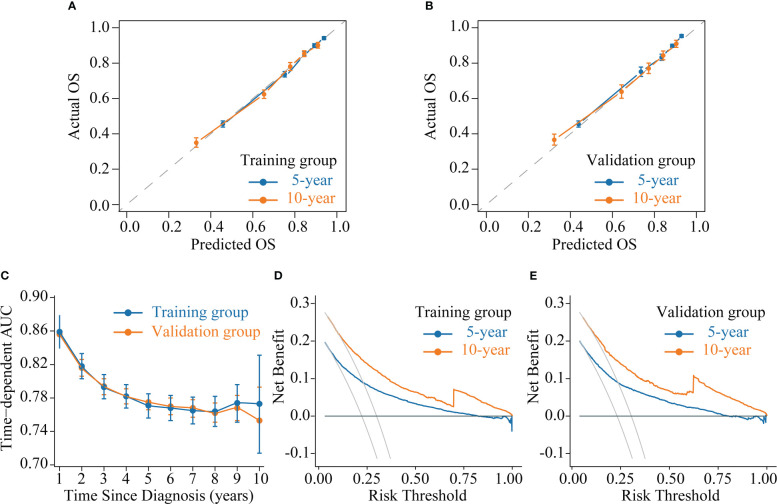
Model evaluation and validation. **(A-B)** Calibration plots, **(C)** Time-dependent area under curve (AUC) curves, and **(D-E)** decision curve analysis (DCA) curves for assessing the accuracy, discrimination and clinical usefulness of the conditional survival nomogram (CS-nomogram).

We evaluated the accuracy of the CS-nomogram in both the training and validation groups through calibration plots, which showed good agreement between the model predictions and the actuals with 5- and 10-year predictive calibration curves that almost coincided with the ideal curve (**Figures 5A, B**). In addition, The C-index was 0.773 (95% CI: 0.765-0.780) and 0.773 (95% CI: 0.761-0.784) for the training and validation groups, respectively, and the time-dependent AUC curves demonstrated that the model’s discrimination did not deteriorate over time, with the stable 10-year AUCs between 0.75 and 0.86 (**Figure 5C**). The DCA curves showed the net benefit of using the CS-nomogram as a tool for triggering medical intervention compared with treating all or nothing all or nothing. The estimated benefit range of the model risk threshold probabilities was 0.08-1.00 and 0.11-1.00 at 5 and 10 years in the training group (**Figure 5D**) and 0.78-1.00 and 0.12-1.00 at 5 and 10 years in the validation group (**Figure 5E**), respectively.

## 4 Discussion

This study was the first to analyze conditional survival patterns in non-metastatic TNBC patients and found that real-time OS in survivors improved dynamically over time. Meanwhile, this improvement was non-linear and slow in the 2nd year after diagnosis. To enable individualized and real-time prognostic assessment, we developed the CS-nomogram, which provided survivors with real-time updated on survival information.

Non-metastatic TNBC is the most malignant subtype of breast cancer but is benefiting from advances in treatment, with patients achieving long-term survival (1–6, 28–30). However, when survivors hope for accurate information about their current prognosis at follow-up, traditional survival analysis may not allow for real-time response (2–7, 28, 31). CS provides a way to quantify the improvement of a survivor’s prognosis over time, making it easier for clinicians and patients to understand. In our study, the survival of non-metastatic TNBC patients did improve over time, and these valuable data helped to reduce patient anxiety levels and improve adherence to cancer follow-up. Second, according to the guideline recommendation, the routine follow-up period for breast cancer was five years. For non-metastatic TNBC patients, it may be seven years, after which survivors’ 10-year real-time OS remained above 95%, meaning they were no different from the general population (32, 33). In addition, we found that patients’ 1-year CS (the probability of surviving several years after diagnosis and then living for one year) decreased in the second year (from CS(1|0) =96.1% to CS(1|1) =93.2%, **Figure 2B**). This result corresponded to a high mortality rate in 2nd year in the annual hazard curve and validated the M.D. Anderson Cancer Center’s conclusion that non-metastatic TNBC might have a higher risk of recurrence and death in the first three years of follow-up (7). The exact cause of the substantial increase in mortality in patients in the 2nd year after diagnosis remained unclear. One explanation was that the intensity of supportive therapy received by patients decreased from the first year onwards, leading to poor survival in the next year. According to the “natural selection” effect of the Zamboni et al. residual lifetime analysis (13), the death of high-risk non-metastatic TNBC patients might promote natural selection in low-risk patients, resulting in a more favorable prognosis for survivors. Therefore, more frequent follow-up over two years was necessary for non-metastatic TNBC, and the follow-up strategy should incorporate the results of CS analysis into the final decision.

Follow-up is a dynamic process, and it is necessary to develop a dynamic assessment tool (14). However, all currently published TNBC nomograms never provided real-time updated prognostic information over time for survivors, for which we developed a novel CS-nomogram. This model may be of more interest to long-term survivors than the traditional nomogram, especially for those with poor initial survival. With both dynamic assessment and personalized prediction, the CS-nomogram provided real-time updated prognostic information over survival time. For example, a patient has a 10-year OS of 35% at the initial diagnosis. After 5 years of follow-up, the CS model can tell her, “Your 10-year survival rate has increased by 40%”, which will significantly strengthen her confidence in fighting cancer. Clinicians can adjust the patient-appropriate monitoring plan in real-time based on the changing risk. The application of the CS-nomogram cannot be limited to non-metastatic TNBC but to all current high-burden diseases. This method will be one of the effective strategies to optimize clinical decision-making to clarify patient prognosis and save healthcare resources.

The CS analysis was calculated as a subset of survivors in the data, which was why we used the SEER database, because the data from surviving patients decreased over time (10, 11). Regarding the performance of the CS-nomogram, the calibration curves nearly overlapping with the ideal curve, the stable AUC curves over 10 years, and the DCA that showed a high net benefit were all evidence of its strength. Thus, this novel model provided prognostic information consistent with real-time follow-up and had considerable clinical utility.

Our study had several limitations. First, as a retrospective study, it was inevitably biased; second, the SEER database was missing variables such as lymphatic vascular invasion, Ki-67, chemotherapy cycles and drugs, and radiotherapy doses, which might limit some of our analyses. Third, our study was unsuitable for patients with metastatic TNBC because their treatment was inconsistent with stage I-III, which may affect the model performance. Fourth, external validation was challenging because of the large amount of data needed for CS analysis. Fifth, the CS nomogram needed to be updated within a few years with the advent of new therapies.

## 5 Conclusion

The CS analysis in this study clarified the dynamic and non-constant improvement in survival over time in non-metastatic TNBC patients. For real-time and personalized prognostic prediction, we developed a novel CS-nomogram. This model provided accurate prognostic information to assist in optimizing clinical decisions and significantly reduced the psychological stress of survivors through real-time prognosis updates. However, future studies need prospective data and more predictors to validate and update the model to make it more generalizable.

## Data availability statement

The raw data supporting the conclusions of this article will be made available by the authors, without undue reservation.

## Ethics statement

Ethical review and approval was not required for the study on human participants in accordance with the local legislation and institutional requirements. The ethics committee waived the requirement of written informed consent for participation.

## Author contributions

XM and YG: Designed the study. XM, YC, and XC: Performed the study and analyzed the data. XM: Wrote the manuscript. XM and YG: Provided the expert consultations and clinical suggestions. XC and YC: Conceived of the study, participated in its design, and coordination. YC, XC, and YG: Helped to draft the manuscript. All authors contributed to the article and approved the submitted version.
