# Supplementary material for: A novel conditional survival nomogram for monitoring real-time prognosis of non-metastatic triple-negative breast cancer
Source: Front Endocrinol (Lausanne). 2023 Feb 24;14:1119105. doi: 10.3389/fendo.2023.1119105 (PMC9998975; doi:10.3389/fendo.2023.1119105)
Supplement: Supplementary file 1 [file Image_1.pdf]

Supplementary Material

A novel conditional survival nomogram for monitoring real-time prognosis of non-metastatic triple-negative breast cancer

Xiangdi Meng [1], Yuanyuan Cai [1], Xiaolong Chang [1], Yinghua Guo [1]*

*** Correspondence:** Yinghua Guo, guoyinghua_wfph@163.com

Supplementary Figures

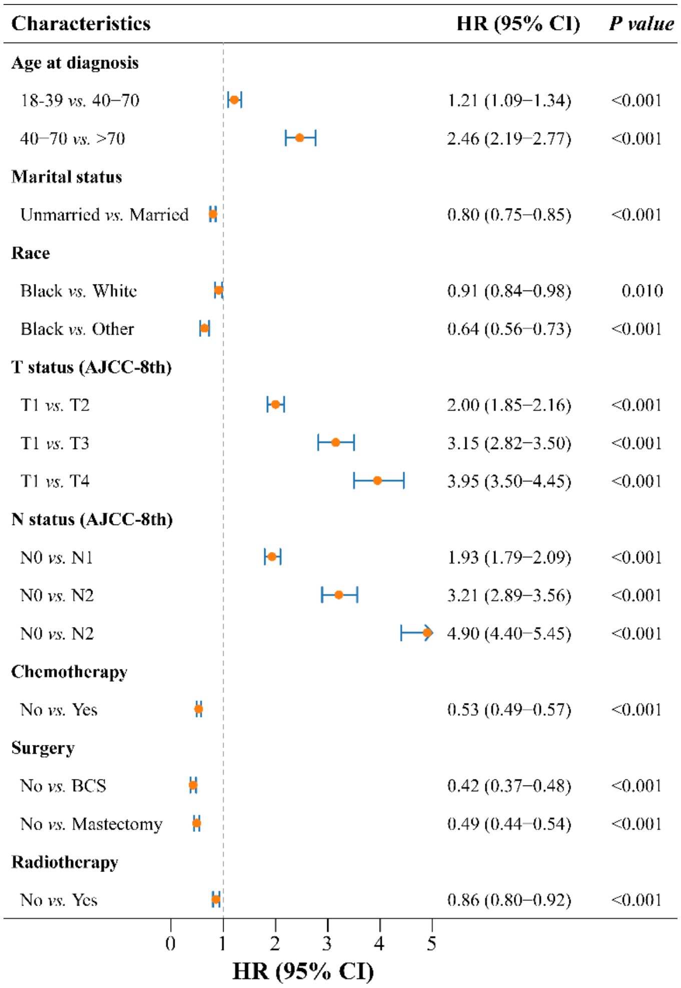

Supplementary Figure 1. Multivariate Cox regression forest plot for revealing the effect of predictors on survival in non-metastatic triple-negative breast cancer. Abbreviations: HR, hazard ratio; CI, confidence interval; AJCC-8th, American Joint Committee on Cancer (8th Edition); BCS, breast conservation surgery
